# (1R*,2R*,3S*,4R*)-Diethyl 4-hy­droxy-4-methyl-2-(4-methyl­phen­yl)-6-oxo­cyclo­hexane-1,3-di­carboxyl­ate

**DOI:** 10.1107/S1600536813009872

**Published:** 2013-04-13

**Authors:** Arif I. Ismiev, Narmina A. Gadirova, Kushvar E. Hajiyeva, Rizvan K. Askerov, Konstantin A. Potekhin

**Affiliations:** a Baku State University, Z. Khalilov St 23, Baku AZ-1148, Azerbaijan; b Vladimir State University, Qor’ky St. 87, Vladimir 600000, Russian Federation

## Abstract

The title compound, C_20_H_26_O_6_, is chiral and crystallizes as a racemate: the relative configuration of the stereogenic centres is 1R*,2R*,3S*,4R*. The cyclo­hexane ring has a chair conformation. The ethyl fragment of the eth­oxy­carbonyl group in the 3-position is disordered over two sets of sites in a 0.650 (6):0.350 (6) ratio. The hy­droxy group acts as a bifurcated hydrogen-bond donor, forming both intra- and inter­molecular hydrogen bonds with ester carbonyl O atoms. The inter­molecular hydrogen bonds form inversion dimers in the crystal.

## Related literature

For applications of related compounds as synthetic inter­mediates, see: Gein *et al.* (2003 ▶, 2004 ▶); Sorokin *et al.* (2000 ▶).
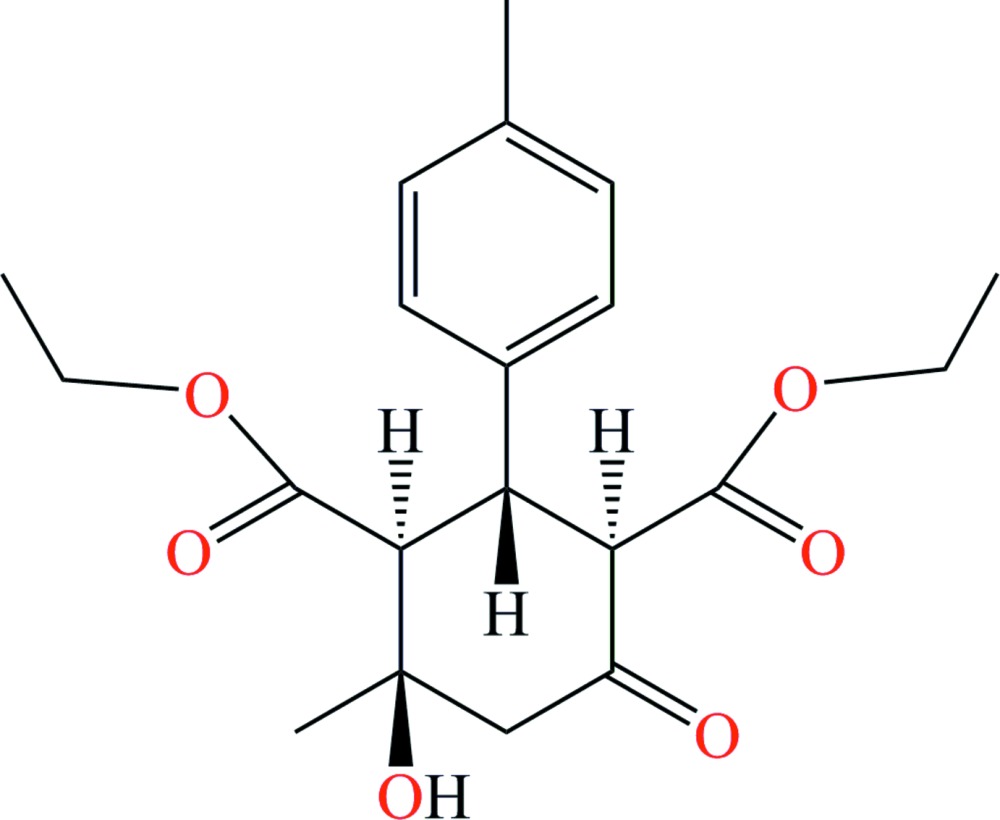


## Experimental

### 

#### Crystal data

C_20_H_26_O_6_
*M* *_r_* = 362.41
Triclinic, 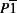

*a* = 5.8062 (4) Å
*b* = 9.9267 (7) Å
*c* = 18.4548 (13) Å
α = 103.281 (2)°
β = 92.490 (2)°
γ = 104.741 (2)°
*V* = 995.26 (12) Å^3^
*Z* = 2
Mo *K*α radiation
μ = 0.09 mm^−1^
*T* = 296 K
0.30 × 0.20 × 0.20 mm

#### Data collection

Bruker APEXII CCD diffractometer
Absorption correction: multi-scan (*SADABS*; Sheldrick, 2003 ▶) *T*_min_ = 0.974, *T*_max_ = 0.983
10589 measured reflections
4344 independent reflections
2735 reflections with *I* > 2σ(*I*)
*R*_int_ = 0.024

#### Refinement

*R*[*F* ^2^ > 2σ(*F* ^2^)] = 0.053
*wR*(*F* ^2^) = 0.141
*S* = 1.01
4344 reflections
245 parameters
4 restraints
H atoms treated by a mixture of independent and constrained refinement
Δρ_max_ = 0.30 e Å^−3^
Δρ_min_ = −0.28 e Å^−3^

### 

Data collection: *APEX2* (Bruker, 2005 ▶); cell refinement: *SAINT-Plus* (Bruker, 2001 ▶); data reduction: *SAINT-Plus*; program(s) used to solve structure: *SHELXTL* (Sheldrick, 2008 ▶); program(s) used to refine structure: *SHELXTL*; molecular graphics: *SHELXTL*; software used to prepare material for publication: *SHELXTL*.

## Supplementary Material

Crystal structure: contains datablock(s) global, I. DOI: 10.1107/S1600536813009872/fy2087sup1.cif

Structure factors: contains datablock(s) I. DOI: 10.1107/S1600536813009872/fy2087Isup2.hkl

Supplementary material file. DOI: 10.1107/S1600536813009872/fy2087Isup3.cml

Additional supplementary materials:  crystallographic information; 3D view; checkCIF report

## Figures and Tables

**Table 1:** Hydrogen-bond geometry (Å, °)

*D*—H⋯*A*	*D*—H	H⋯*A*	*D*⋯*A*	*D*—H⋯*A*
O6—H6⋯O1	0.91 (4)	2.28 (4)	2.884 (2)	124 (3)
O6—H6⋯O1^i^	0.91 (4)	2.28 (4)	3.066 (2)	145 (3)

Symmetry code: (i) 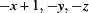.

**Fig. 1.**
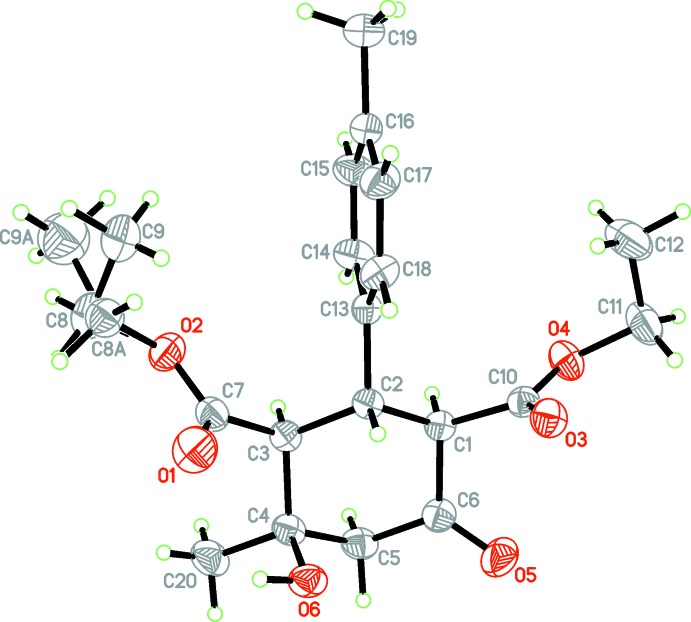
Molecular structure of the title compound with the atom numbering scheme. Displacement ellipsoids were drawn at the 30% probability level.

**Fig. 2.**
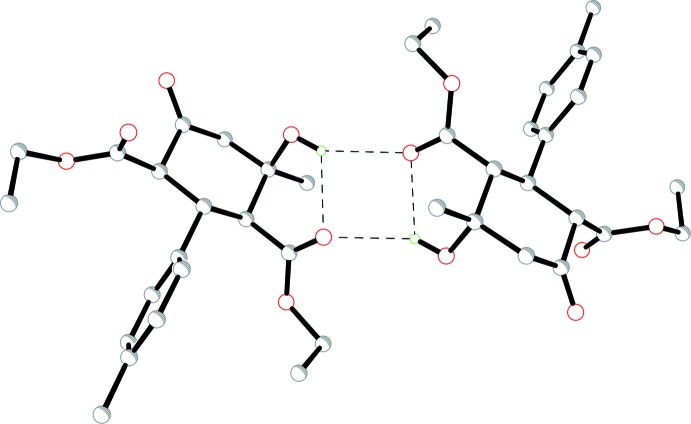
Perspective view of the dimer. H bonds are shown as a dashed lines. H atoms that not involved in the formation of hydrogen bonds are not shown.

**Fig. 3.**
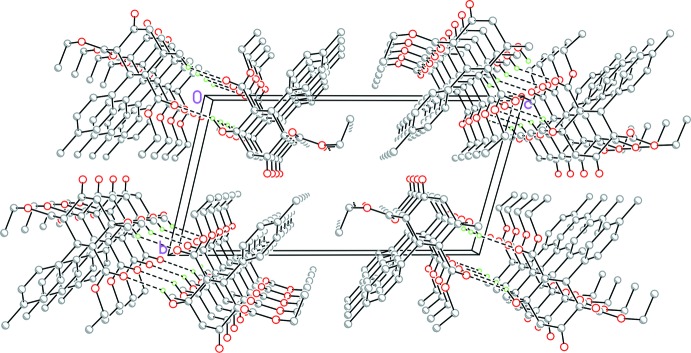
Stacking of the dimers in the crystal lattice.

## supplementary crystallographic information

### Comment

The relative arrangement of functional groups in polyfunctional diethoxycarbonyl substituted cyclohexane β-ketols renders them favorable intermediates for the construction of enamines and nitrogen heterocyclic compounds (Gein *et al.*, 2003; Gein *et al.*, 2004; Sorokin *et al.*, 2000). From our point of view, it is important to determine the molecular crystal structure of the initial β-ketols to aid the exploration of heterocyclization reactions.

Fig. 1 shows the molecular structure of title compound (I) C_20_H_26_O_6_. The cyclohexane ring has a chair conformation (Fig. 2). Four atoms of the cyclohexane ring, C2, C3, C5 and C6 are located on the same plane [r.m.s deviation = 0.011 (2) Å], while C1 [deviation = 0.617 (2) Å] and C4 [deviation = -0.698 (2) Å] deviate from the plane. The ethyl fragment of the ethoxycarbonyl group at the position of C3 is disordered over two sets of sites in a 0.650 (6):0.350 (6) ratio. The OH group participates in the formation of both intramolecular and intermolecular hydrogen bonds. The intermolecular hydrogen bonds form centrosymmetric dimers. These dimers form stacks in the direction of the axis *a* (Fig. 3). The stacking of dimers is governed by van der Waals interactions.

### Experimental

4-methyl benzaldehyde (10 mmol) and acetoacetic ester (20 mmol) were dissolved in 20 ml ethanol. 1 ml piperidine was added to the reaction mixture. After 48 h the obtained crystals were filtered and washed with ethanol. The crystals were dissolved in ethanol (50 ml) and recrystallized by slow solvent evaporation to yield the crystals of the title compound suitable for X-ray analysis.

### Refinement

The hydrogen atom of the OH group was found in a difference Fourier map and was included in the refinement with isotropic displacement parameters. The other hydrogen atoms were placed in calculated positions with C—H distances of 0.93–0.98 Å and were refined in the riding mode with fixed isotropic displacement parameters [*U*_iso_(H) = 1.2 *U*_eq_(C) or 1.5*U*_eq_(C) for methyl H atoms]. The C—C and C—O bond distances of the disordered ethoxy group were restrained to 1.540 (4) and 1.453 (4) Å, respectively.

### Crystal data

**Table:**

C_20_H_26_O_6_	*Z* = 2
*M**_r_* = 362.41	*F*(000) = 388
Triclinic, *P*1	*D*_x_ = 1.209 Mg m^−^^3^
Hall symbol: -P 1	Mo *K*α radiation, λ = 0.71073 Å
*a* = 5.8062 (4) Å	Cell parameters from 2311 reflections
*b* = 9.9267 (7) Å	θ = 2.2–24.0°
*c* = 18.4548 (13) Å	µ = 0.09 mm^−^^1^
α = 103.281 (2)°	*T* = 296 K
β = 92.490 (2)°	Prism, colourless
γ = 104.741 (2)°	0.30 × 0.20 × 0.20 mm
*V* = 995.26 (12) Å^3^	

### Data collection

**Table:**

Bruker APEXII CCD diffractometer	4344 independent reflections
Radiation source: fine-focus sealed tube	2735 reflections with *I* > 2σ(*I*)
Graphite monochromator	*R*_int_ = 0.024
φ and ω scans	θ_max_ = 27.0°, θ_min_ = 2.2°
Absorption correction: multi-scan (*SADABS*; Sheldrick, 2003)	*h* = −7→7
*T*_min_ = 0.974, *T*_max_ = 0.983	*k* = −12→12
10589 measured reflections	*l* = −23→23

### Refinement

**Table:**

Refinement on *F*^2^	Primary atom site location: structure-invariant direct methods
Least-squares matrix: full	Secondary atom site location: difference Fourier map
*R*[*F*^2^ > 2σ(*F*^2^)] = 0.053	Hydrogen site location: difference Fourier map
*wR*(*F*^2^) = 0.141	H atoms treated by a mixture of independent and constrained refinement
*S* = 1.01	*w* = 1/[σ^2^(*F*_o_^2^) + (0.0532*P*)^2^ + 0.330*P*] where *P* = (*F*_o_^2^ + 2*F*_c_^2^)/3
4344 reflections	(Δ/σ)_max_ < 0.001
245 parameters	Δρ_max_ = 0.30 e Å^−^^3^
4 restraints	Δρ_min_ = −0.28 e Å^−^^3^

### Special details

**Table:**

Geometry. All e.s.d.'s (except the e.s.d. in the dihedral angle between two l.s. planes) are estimated using the full covariance matrix. The cell e.s.d.'s are taken into account individually in the estimation of e.s.d.'s in distances, angles and torsion angles; correlations between e.s.d.'s in cell parameters are only used when they are defined by crystal symmetry. An approximate (isotropic) treatment of cell e.s.d.'s is used for estimating e.s.d.'s involving l.s. planes.
Refinement. Refinement of *F*^2^ against ALL reflections. The weighted *R*-factor *wR* and goodness of fit *S* are based on *F*^2^, conventional *R*-factors *R* are based on *F*, with *F* set to zero for negative *F*^2^. The threshold expression of *F*^2^ > σ(*F*^2^) is used only for calculating *R*-factors(gt) *etc*. and is not relevant to the choice of reflections for refinement. *R*-factors based on *F*^2^ are statistically about twice as large as those based on *F*, and *R*- factors based on ALL data will be even larger.

### Fractional atomic coordinates and isotropic or equivalent isotropic displacement parameters (Å^2^)

**Table:**

	*x*	*y*	*z*	*U*_iso_*/*U*_eq_	Occ. (<1)
O1	0.4611 (3)	−0.07429 (19)	0.06812 (9)	0.0807 (6)	
O2	0.1412 (3)	−0.13134 (16)	0.12814 (9)	0.0695 (5)	
O3	1.0827 (2)	0.24372 (16)	0.31384 (8)	0.0568 (4)	
O4	0.8797 (3)	0.31533 (17)	0.40878 (8)	0.0611 (4)	
O5	0.8906 (3)	0.48221 (18)	0.26673 (11)	0.0808 (6)	
O6	0.6651 (3)	0.23247 (18)	0.09792 (9)	0.0554 (4)	
H6	0.628 (6)	0.154 (4)	0.058 (2)	0.131 (14)*	
C1	0.6833 (3)	0.2564 (2)	0.28710 (10)	0.0416 (4)	
H1A	0.5523	0.2588	0.3187	0.050*	
C2	0.6142 (3)	0.10970 (19)	0.22851 (10)	0.0394 (4)	
H2A	0.7510	0.1038	0.1998	0.047*	
C3	0.4002 (3)	0.1013 (2)	0.17338 (11)	0.0434 (5)	
H3A	0.2620	0.1059	0.2015	0.052*	
C4	0.4528 (3)	0.2273 (2)	0.13498 (11)	0.0476 (5)	
C5	0.5117 (4)	0.3687 (2)	0.19497 (12)	0.0544 (5)	
H5A	0.5521	0.4481	0.1713	0.065*	
H5B	0.3715	0.3751	0.2208	0.065*	
C6	0.7152 (4)	0.3814 (2)	0.25046 (12)	0.0505 (5)	
C7	0.3412 (4)	−0.0423 (2)	0.11719 (12)	0.0518 (5)	
C8	0.0451 (13)	−0.2768 (4)	0.0822 (3)	0.1013 (17)	0.650 (6)
H8A	0.0897	−0.2839	0.0317	0.122*	0.650 (6)
H8B	−0.1283	−0.3060	0.0800	0.122*	0.650 (6)
C9	0.1545 (10)	−0.3702 (5)	0.1203 (3)	0.1013 (17)	0.650 (6)
H9A	0.0983	−0.4686	0.0922	0.152*	0.650 (6)
H9B	0.1082	−0.3618	0.1701	0.152*	0.650 (6)
H9C	0.3259	−0.3389	0.1227	0.152*	0.650 (6)
C8A	0.1229 (14)	−0.2769 (5)	0.0834 (5)	0.062 (3)*	0.350 (6)
H8AA	0.2699	−0.3026	0.0926	0.074*	0.350 (6)
H8AB	0.0984	−0.2809	0.0305	0.074*	0.350 (6)
C9A	−0.0872 (18)	−0.3816 (10)	0.1050 (6)	0.114 (4)*	0.350 (6)
H9AA	−0.0989	−0.4776	0.0767	0.171*	0.350 (6)
H9AB	−0.2325	−0.3570	0.0945	0.171*	0.350 (6)
H9AC	−0.0626	−0.3761	0.1575	0.171*	0.350 (6)
C10	0.9053 (4)	0.2708 (2)	0.33654 (11)	0.0439 (5)	
C11	1.0701 (5)	0.3137 (3)	0.46185 (14)	0.0761 (8)	
H11A	1.0708	0.3809	0.5093	0.091*	
H11B	1.2236	0.3434	0.4431	0.091*	
C12	1.0331 (6)	0.1658 (3)	0.47313 (16)	0.0921 (9)	
H12A	1.1512	0.1671	0.5115	0.138*	
H12B	1.0485	0.1014	0.4272	0.138*	
H12C	0.8760	0.1339	0.4879	0.138*	
C13	0.5667 (3)	−0.01238 (19)	0.26723 (10)	0.0394 (4)	
C14	0.3813 (4)	−0.0336 (2)	0.31157 (12)	0.0556 (6)	
H14A	0.2828	0.0279	0.3177	0.067*	
C15	0.3402 (4)	−0.1444 (3)	0.34675 (13)	0.0616 (6)	
H15A	0.2136	−0.1565	0.3759	0.074*	
C16	0.4826 (4)	−0.2382 (2)	0.33975 (12)	0.0537 (5)	
C17	0.6670 (4)	−0.2166 (2)	0.29575 (13)	0.0583 (6)	
H17A	0.7665	−0.2775	0.2901	0.070*	
C18	0.7080 (4)	−0.1061 (2)	0.25972 (12)	0.0504 (5)	
H18A	0.8332	−0.0950	0.2299	0.060*	
C19	0.4324 (5)	−0.3606 (3)	0.37778 (15)	0.0763 (8)	
H19A	0.5673	−0.4000	0.3769	0.114*	
H19B	0.2928	−0.4339	0.3519	0.114*	
H19C	0.4052	−0.3255	0.4288	0.114*	
C20	0.2422 (4)	0.2159 (3)	0.07980 (14)	0.0680 (7)	
H20A	0.2770	0.2975	0.0585	0.102*	
H20B	0.1014	0.2137	0.1054	0.102*	
H20C	0.2152	0.1293	0.0407	0.102*	

### Atomic displacement parameters (Å^2^)

**Table:**

	*U*^11^	*U*^22^	*U*^33^	*U*^12^	*U*^13^	*U*^23^
O1	0.0936 (13)	0.0774 (12)	0.0587 (10)	0.0147 (10)	0.0294 (10)	−0.0019 (9)
O2	0.0692 (11)	0.0619 (10)	0.0632 (10)	−0.0041 (8)	0.0070 (8)	0.0117 (8)
O3	0.0440 (8)	0.0673 (10)	0.0644 (10)	0.0212 (7)	0.0064 (7)	0.0197 (8)
O4	0.0712 (10)	0.0678 (10)	0.0452 (8)	0.0292 (8)	−0.0008 (7)	0.0059 (7)
O5	0.0744 (12)	0.0503 (10)	0.1131 (15)	0.0027 (9)	−0.0191 (10)	0.0327 (10)
O6	0.0469 (8)	0.0681 (10)	0.0568 (9)	0.0153 (7)	0.0161 (7)	0.0252 (8)
C1	0.0396 (10)	0.0436 (11)	0.0444 (11)	0.0158 (8)	0.0069 (9)	0.0115 (9)
C2	0.0366 (10)	0.0442 (11)	0.0411 (10)	0.0150 (8)	0.0085 (8)	0.0125 (8)
C3	0.0371 (10)	0.0528 (12)	0.0425 (11)	0.0145 (9)	0.0088 (8)	0.0132 (9)
C4	0.0410 (11)	0.0609 (13)	0.0480 (11)	0.0189 (9)	0.0079 (9)	0.0214 (10)
C5	0.0567 (13)	0.0541 (13)	0.0635 (14)	0.0258 (10)	0.0082 (11)	0.0244 (11)
C6	0.0521 (12)	0.0430 (12)	0.0590 (13)	0.0179 (10)	0.0044 (10)	0.0123 (10)
C7	0.0535 (13)	0.0589 (13)	0.0417 (11)	0.0127 (11)	0.0041 (10)	0.0132 (10)
C8	0.124 (4)	0.071 (2)	0.085 (3)	−0.003 (2)	−0.022 (2)	0.0108 (17)
C9	0.124 (4)	0.071 (2)	0.085 (3)	−0.003 (2)	−0.022 (2)	0.0108 (17)
C10	0.0479 (12)	0.0353 (10)	0.0490 (12)	0.0122 (9)	0.0044 (9)	0.0105 (9)
C11	0.095 (2)	0.0757 (18)	0.0550 (14)	0.0293 (15)	−0.0175 (14)	0.0088 (13)
C12	0.127 (3)	0.094 (2)	0.0652 (17)	0.0392 (19)	−0.0012 (17)	0.0311 (16)
C13	0.0374 (10)	0.0405 (10)	0.0405 (10)	0.0121 (8)	0.0039 (8)	0.0087 (8)
C14	0.0566 (13)	0.0656 (14)	0.0604 (13)	0.0299 (11)	0.0210 (11)	0.0293 (11)
C15	0.0578 (14)	0.0756 (16)	0.0618 (14)	0.0191 (12)	0.0189 (11)	0.0344 (13)
C16	0.0566 (13)	0.0474 (12)	0.0530 (13)	0.0046 (10)	−0.0064 (11)	0.0175 (10)
C17	0.0549 (13)	0.0462 (12)	0.0780 (16)	0.0190 (10)	0.0040 (12)	0.0183 (11)
C18	0.0463 (11)	0.0440 (11)	0.0630 (13)	0.0146 (9)	0.0134 (10)	0.0138 (10)
C19	0.0884 (19)	0.0631 (16)	0.0767 (17)	0.0067 (14)	−0.0060 (15)	0.0347 (14)
C20	0.0573 (14)	0.0904 (18)	0.0661 (15)	0.0255 (13)	0.0000 (12)	0.0339 (14)

### Geometric parameters (Å, º)

**Table:**

O1—C7	1.200 (2)	C9—H9C	0.9600
O2—C7	1.323 (3)	C8A—C9A	1.522 (4)
O2—C8	1.449 (3)	C8A—H8AA	0.9700
O2—C8A	1.465 (4)	C8A—H8AB	0.9700
O3—C10	1.198 (2)	C9A—H9AA	0.9600
O4—C10	1.332 (2)	C9A—H9AB	0.9600
O4—C11	1.450 (3)	C9A—H9AC	0.9600
O5—C6	1.203 (3)	C11—C12	1.493 (4)
O6—C4	1.431 (2)	C11—H11A	0.9700
O6—H6	0.91 (4)	C11—H11B	0.9700
C1—C10	1.503 (3)	C12—H12A	0.9600
C1—C6	1.520 (3)	C12—H12B	0.9600
C1—C2	1.547 (3)	C12—H12C	0.9600
C1—H1A	0.9800	C13—C18	1.377 (3)
C2—C13	1.517 (3)	C13—C14	1.383 (3)
C2—C3	1.544 (3)	C14—C15	1.377 (3)
C2—H2A	0.9800	C14—H14A	0.9300
C3—C7	1.505 (3)	C15—C16	1.382 (3)
C3—C4	1.546 (3)	C15—H15A	0.9300
C3—H3A	0.9800	C16—C17	1.375 (3)
C4—C20	1.521 (3)	C16—C19	1.511 (3)
C4—C5	1.524 (3)	C17—C18	1.385 (3)
C5—C6	1.490 (3)	C17—H17A	0.9300
C5—H5A	0.9700	C18—H18A	0.9300
C5—H5B	0.9700	C19—H19A	0.9600
C8—C9	1.522 (4)	C19—H19B	0.9600
C8—H8A	0.9700	C19—H19C	0.9600
C8—H8B	0.9700	C20—H20A	0.9600
C9—H9A	0.9600	C20—H20B	0.9600
C9—H9B	0.9600	C20—H20C	0.9600
			
C7—O2—C8	123.5 (4)	O2—C8A—H8AB	109.9
C7—O2—C8A	109.3 (3)	C9A—C8A—H8AB	109.9
C10—O4—C11	116.33 (18)	H8AA—C8A—H8AB	108.3
C4—O6—H6	106 (2)	C8A—C9A—H9AA	109.5
C10—C1—C6	111.07 (16)	C8A—C9A—H9AB	109.5
C10—C1—C2	110.21 (14)	H9AA—C9A—H9AB	109.5
C6—C1—C2	111.87 (16)	C8A—C9A—H9AC	109.5
C10—C1—H1A	107.8	H9AA—C9A—H9AC	109.5
C6—C1—H1A	107.8	H9AB—C9A—H9AC	109.5
C2—C1—H1A	107.8	O3—C10—O4	124.06 (19)
C13—C2—C3	113.00 (15)	O3—C10—C1	124.13 (18)
C13—C2—C1	110.32 (15)	O4—C10—C1	111.80 (17)
C3—C2—C1	109.93 (14)	O4—C11—C12	109.8 (2)
C13—C2—H2A	107.8	O4—C11—H11A	109.7
C3—C2—H2A	107.8	C12—C11—H11A	109.7
C1—C2—H2A	107.8	O4—C11—H11B	109.7
C7—C3—C2	107.99 (16)	C12—C11—H11B	109.7
C7—C3—C4	111.78 (16)	H11A—C11—H11B	108.2
C2—C3—C4	111.96 (15)	C11—C12—H12A	109.5
C7—C3—H3A	108.3	C11—C12—H12B	109.5
C2—C3—H3A	108.3	H12A—C12—H12B	109.5
C4—C3—H3A	108.3	C11—C12—H12C	109.5
O6—C4—C20	110.23 (17)	H12A—C12—H12C	109.5
O6—C4—C5	104.60 (17)	H12B—C12—H12C	109.5
C20—C4—C5	110.88 (18)	C18—C13—C14	117.48 (18)
O6—C4—C3	110.68 (15)	C18—C13—C2	121.02 (17)
C20—C4—C3	111.32 (17)	C14—C13—C2	121.50 (16)
C5—C4—C3	108.92 (16)	C15—C14—C13	121.1 (2)
C6—C5—C4	111.90 (16)	C15—C14—H14A	119.5
C6—C5—H5A	109.2	C13—C14—H14A	119.5
C4—C5—H5A	109.2	C14—C15—C16	121.7 (2)
C6—C5—H5B	109.2	C14—C15—H15A	119.2
C4—C5—H5B	109.2	C16—C15—H15A	119.2
H5A—C5—H5B	107.9	C17—C16—C15	117.14 (19)
O5—C6—C5	123.9 (2)	C17—C16—C19	121.9 (2)
O5—C6—C1	121.56 (19)	C15—C16—C19	120.9 (2)
C5—C6—C1	114.57 (18)	C16—C17—C18	121.5 (2)
O1—C7—O2	123.2 (2)	C16—C17—H17A	119.3
O1—C7—C3	124.5 (2)	C18—C17—H17A	119.3
O2—C7—C3	112.31 (18)	C13—C18—C17	121.2 (2)
O2—C8—C9	105.3 (3)	C13—C18—H18A	119.4
O2—C8—H8A	110.7	C17—C18—H18A	119.4
C9—C8—H8A	110.7	C16—C19—H19A	109.5
O2—C8—H8B	110.7	C16—C19—H19B	109.5
C9—C8—H8B	110.7	H19A—C19—H19B	109.5
H8A—C8—H8B	108.8	C16—C19—H19C	109.5
C8—C9—H9A	109.5	H19A—C19—H19C	109.5
C8—C9—H9B	109.5	H19B—C19—H19C	109.5
H9A—C9—H9B	109.5	C4—C20—H20A	109.5
C8—C9—H9C	109.5	C4—C20—H20B	109.5
H9A—C9—H9C	109.5	H20A—C20—H20B	109.5
H9B—C9—H9C	109.5	C4—C20—H20C	109.5
O2—C8A—C9A	108.8 (6)	H20A—C20—H20C	109.5
O2—C8A—H8AA	109.9	H20B—C20—H20C	109.5
C9A—C8A—H8AA	109.9		
			
C10—C1—C2—C13	59.94 (19)	C4—C3—C7—O1	51.1 (3)
C6—C1—C2—C13	−175.97 (15)	C2—C3—C7—O2	106.96 (19)
C10—C1—C2—C3	−174.79 (15)	C4—C3—C7—O2	−129.45 (18)
C6—C1—C2—C3	−50.7 (2)	C7—O2—C8—C9	90.5 (5)
C13—C2—C3—C7	−56.8 (2)	C8A—O2—C8—C9	50.2 (14)
C1—C2—C3—C7	179.47 (16)	C7—O2—C8A—C9A	171.7 (6)
C13—C2—C3—C4	179.72 (15)	C8—O2—C8A—C9A	−43.2 (13)
C1—C2—C3—C4	56.0 (2)	C11—O4—C10—O3	−7.4 (3)
C7—C3—C4—O6	−65.3 (2)	C11—O4—C10—C1	171.67 (18)
C2—C3—C4—O6	56.0 (2)	C6—C1—C10—O3	−77.4 (2)
C7—C3—C4—C20	57.6 (2)	C2—C1—C10—O3	47.1 (3)
C2—C3—C4—C20	178.94 (17)	C6—C1—C10—O4	103.56 (19)
C7—C3—C4—C5	−179.81 (16)	C2—C1—C10—O4	−131.89 (17)
C2—C3—C4—C5	−58.5 (2)	C10—O4—C11—C12	−82.2 (3)
O6—C4—C5—C6	−62.1 (2)	C3—C2—C13—C18	120.07 (19)
C20—C4—C5—C6	179.10 (18)	C1—C2—C13—C18	−116.4 (2)
C3—C4—C5—C6	56.3 (2)	C3—C2—C13—C14	−60.3 (2)
C4—C5—C6—O5	127.0 (2)	C1—C2—C13—C14	63.3 (2)
C4—C5—C6—C1	−54.1 (2)	C18—C13—C14—C15	0.0 (3)
C10—C1—C6—O5	−6.5 (3)	C2—C13—C14—C15	−179.7 (2)
C2—C1—C6—O5	−130.1 (2)	C13—C14—C15—C16	0.4 (4)
C10—C1—C6—C5	174.63 (17)	C14—C15—C16—C17	−0.3 (3)
C2—C1—C6—C5	51.0 (2)	C14—C15—C16—C19	−179.0 (2)
C8—O2—C7—O1	0.0 (4)	C15—C16—C17—C18	−0.3 (3)
C8A—O2—C7—O1	12.1 (5)	C19—C16—C17—C18	178.5 (2)
C8—O2—C7—C3	−179.5 (3)	C14—C13—C18—C17	−0.5 (3)
C8A—O2—C7—C3	−167.4 (4)	C2—C13—C18—C17	179.17 (19)
C2—C3—C7—O1	−72.5 (3)	C16—C17—C18—C13	0.7 (3)

### Hydrogen-bond geometry (Å, º)

**Table:**

*D*—H···*A*	*D*—H	H···*A*	*D*···*A*	*D*—H···*A*
O6—H6···O1	0.91 (4)	2.28 (4)	2.884 (2)	124 (3)
O6—H6···O1^i^	0.91 (4)	2.28 (4)	3.066 (2)	145 (3)

Symmetry code: (i) −*x*+1, −*y*, −*z*.
